# Chinese text dual attention network for aspect-level sentiment classification

**DOI:** 10.1371/journal.pone.0295331

**Published:** 2024-03-07

**Authors:** Xinjie Sun, Zhifang Liu, Hui Li, Feng Ying, Yu Tao

**Affiliations:** 1 Institute of Computer Science, Liupanshui Normal University, Liupanshui, Guizhou, China; 2 Guizhou Huohua Technology Co., Ltd, Liupanshui, Guizhou, China; Sunway University, MALAYSIA

## Abstract

English text has a clear and compact subject structure, which makes it easy to find dependency relationships between words. However, Chinese text often conveys information using situational settings, which results in loose sentence structures, and even most Chinese comments and experimental summary texts lack subjects. This makes it challenging to determine the dependency relationship between words in Chinese text, especially in aspect-level sentiment recognition. To solve this problem faced by Chinese text in the field of sentiment recognition, a Chinese text dual attention network for aspect-level sentiment recognition is proposed. First, Chinese syntactic dependency is proposed, and sentiment dictionary is introduced to quickly and accurately extract aspect-level sentiment words, opinion extraction and classification of sentimental trends in text. Additionally, in order to extract context-level features, the CNN-BILSTM model and position coding are also introduced. Finally, to better extract fine-grained aspect-level sentiment, a two-level attention mechanism is used. Compared with ten advanced baseline models, the model’s capabilities are being further optimized for better performance, with Accuracy of 0.9180, 0.9080 and 0.8380 respectively. This method is being demonstrated by a vast array of experiments to achieve higher performance in aspect-level sentiment recognition in less time, and ablation experiments demonstrate the importance of each module of the model.

## 1 Introduction

With the increasing popularity of online education in China, more and more educational datasets are being recorded. On online education platforms, almost all students post comments and upload their homework, including course learning summaries and experiment summaries. These datasets contain both knowledge point descriptions and possible sentiment expressions related to the teaching and mastery of the course. A large number of sentimental data can be found in these online text datasets [1]. An essential area of Natural Language Processing (NLP) [2], is sentiment recognition, which involves the categorization of text according to its sentimental tone or subjective feeling, the primary objective of sentiment recognition is to analyze the sentimental inclination conveyed in brief subjective texts and determine their sentiment trends.

Aspect Based Sentiment Analysis (ABSA) is a highly detailed subfield of sentiment recognition that concentrates on detecting the different elements present in a text and their corresponding sentimental states, including positive, negative, or neutral [3]. This type of analysis aims to accurately express the outline of sentimental information by analyzing the aspect-level sentiment triad, which comprises the aspect word, corresponding opinion word, and corresponding sentiment trend [4]. For instance, the comment “I don’t like database, but the teacher is cute.” contains two aspects of sentimental words, namely “database” and “teacher.” The corresponding opinion words are “don’t like” and “cute,” respectively, and the negative sentiment and positive sentiment are assigned to the “database” and “teacher” corresponding sentimental trends.

Convolutional Neural Networks (CNNs) [5] and Long Short-Term Memory (LSTM) networks [6] are significant models utilized for addressing sentiment recognition challenges. These models are designed to continually acquire new features based on the sequence of the context within the text, but they do not take into account the interrelationship between individual words. To address this limitation, the attention mechanism was proposed in literature [7], which more effectively correlates the words in sentences. Due to the effectiveness of the attention mechanism, BERT [53] is frequently employed for sentiment classification. One approach involves integrating the encoding structure of BERT into other models as an embedding layer, while the other involves directly utilizing BERT for aspect-level sentiment classification. Both methods have been shown to enhance results. As the corresponding sentimental trends of aspect words and their corresponding opinion words are dependent on each other, the utilization of the attention mechanism can assist in determining these trends.

Performing sentiment analysis at the aspect-level, the dependent tree-based Graph Neural Network (GNN) has gained significant attention as a promising area of research [8–10]. The information interaction between different words in the GNN is enhanced in literature [11, 12], whereby syntactic information is utilized to obtain sentence features. In addition, it has been shown to enhance the precision of sentiment recognition of textual data. By employing graph-based techniques, it becomes possible to capture both the semantic and syntactic connections between individual words, thereby enabling a more comprehensive comprehension of the sentiment conveyed within a particular text.

Although good results have been achieved by the above methods, there are still some problems that need to be addressed [9]. Firstly, the contextual information in the original sentences tends to be overlooked by the model that integrates both syntactic and semantic information. Secondly, good results for complex statements are difficult to achieve, it is worth noting that the efficacy of these models is significantly impacted by the precision and correctness of the outputs generated through syntactic resolution. For example, there is such a comment: “Who dares to compare service with big brands, right?” Most models will classify the sentiment towards a service as positive. However, in reality, this evaluation of the service often contains elements of sarcasm, which is inherently negative. Additionally, for sentiment datasets accumulated in online learning platforms, many course summaries and experimental summaries are composed of long texts with complex sentiments, and the relatively loose Chinese grammar further decreases the accuracy of sentiment recognition.

Additionally, a new aspect-level sentiment analysis network (ASN-CSD) is proposed to address the issue of the lack of sentimental knowledge assistance in existing models. The proposed network integrates context-level and sentiment dictionary to fully utilize syntactic dependency and obtain contextual feature information. Firstly, a pre-trained Word2vec model is used to obtain sentence encoding. Secondly, a sentiment dictionary-based syntactic dependency analysis algorithm is introduced to identify aspect-level sentiment information accurately and quickly. Through the utilization of attention mechanisms, the aspect-level sentiment words are intrinsically interconnected, and the model is subsequently fine-tuned accordingly to optimize performance. Thirdly, to extract bidirectional characteristics from the entirety of the sentence, a bidirectional Long Short-Term Memory (LSTM) network is employed, by incorporating attention mechanisms, it becomes feasible to grasp the interconnections between words, thereby enabling a more comprehensive understanding of the relationships between them. Finally, the complete context sentence features and aspect-level sentiment features are concatenated to allow various characteristic parts to influence each other and be mutually beneficial. The proposal of the ASN-CSD framework is the primary contribution of this article, which integrates context-level, sentiment dictionary and effectively utilizes syntactic dependency to obtain contextual feature information for the analysis of sentiment at an aspect-level. Outlined below are the specific contributions of this research.

(1) A sentiment dictionary is developed by incorporating the interdependence relationships among the words present in a given sentence. Additionally, a network model is designed to accurately and efficiently identify the sentiment words present in sentences along with their corresponding sentiment trends (DDP-ASDN).
(2) A novel approach to the analysis of sentiment at an aspect-level is presented, which utilizes a dual attention network. The network incorporates both the contextual information of the complete sentence and the aspect-level sentiment dyads of DDP-ASDN. This model achieves a local-to-holistic combination of information.
(3) The effectiveness of the model is assessed using two publicly available Chinese datasets and a collection of Chinese dataset from the “U+ Wisdom Teaching Platform”. Experimental results demonstrate that the model achieves improved Accuracy, Efficiency, and Macro-average Macro-F1 on different datasets. The importance of the dual attention framework in fusing context and sentiment aspects is further highlighted by these results.

The following is the structure of the article: Section 1, the most recent study on fine-grained the analysis of sentiment at an aspect-level is introduced. Section 2, the latest techniques for both sentiment recognition and aspect-level sentiment recognition are presented separately. Section 3, describes the architecture of the artificial neural network recommended in this article. Section 4, showcases the datasets and experimental results used in the context of this research. Finally, Section 5, a summary of the article’s key takeaways is presented in its conclusion.

## 2 Related work

### 2.1 Sentiment classification

Sentiment classification based on the artificial neural network mainly endeavors to confirm the feature distance of sentiment in a short text by actively updating the weight relationship of the word vectors, and finally trains the optimal feature distance under the model [13]. Sentiment recognition is often accomplished through the use of Convolutional Neural Networks (CNN) [14, 15], but when the data dependence is relatively high, CNN often does not perform well enough due to the lack of internal dependence mechanism. Recurrent Neural Networks (RNN) [16, 17] can handle short-term dependence. However, vanishing and blowing up of gradients are often observed when dealing with long-term dependencies in text. Long Short-Term Memory (LSTM) networks and Bi-directional Long Short-Term Memory (BILSTM) networks [18, 19] has the ability to overcome the challenges of long short-term dependencies to a certain extent, but gradient disappearance still occurs when the text dependence length exceeds a certain limit. Attention mechanisms [20] can obtain the dependence between local and global, compared with CNN and RNN, have few parameters and low model complexity. However, they cannot effectively learn the location information in the text.

Using a single model alone has certain disadvantages. Currently, the mainstream research direction is to stack and modify models, which can help overcome some of these issues. For example, Yang et al. [21] implemented text classification by weighting a modified RNN and combining it with ATTENTION. Similarly, the design of a BILSTM structure incorporating attention is attributed by Wang et al. [22], that can give priority to various segments of the sentence. Dragoni et al. [23] used a neural word embedding approach combined with conventional network models to address the shortcomings of earlier techniques.

The combination of word embedding models and neural network models for sentiment recognition is a significant area of research [24]. For example, Liang et al. [25] employed a pre-trained word2vec structure for word embedding and used a CNN to continuously learn sentence Characteristics and classify sentiments with the aid of the attention mechanism. Meanwhile, Tang et al. [26] utilized a pre-trained Word2vec structure and employed a LSTM to acquire long short-term associations of the text, and then integrated this with an attention mechanism to classify sentiment.

### 2.2 Aspect-level sentiment classification

#### 2.2.1 Method based on sentiment dictionary

The sentiment dictionary constructed on technique utilizes annotated sentiment trend words to classify the sentiment category. First introduced by Whissell et al. [27], early sentimental dictionaries were mainly available in English, such as SentiWordNet can be download from: http://sentiwordnet.isti.cnr.it/. NTUSD can be download from: http://academiasinicanlplab.github.io/. ANTUSD, and HowNet can be download from: http://openhownet.thunlp.org/download. Liang et al. [28] developed a “Chinese sentimental vocabulary ontology library” can be download from: http://ir.dlut.edu.cn/info/1013/1142.htm. Ding et al. [29] recommended a comprehensive sentiment analysis algorithm that utilizes contextual sentiment words and evaluates the distance between sentiment words and aspects. The method assigns different weights to sentimental words and advances the precision of aspect-level sentiment recognition. Xu et al. [30] combined an enriched sentiment dictionary encompassing a larger number of sentimental words with a redesigned sentiment recognition rule based on the dictionary. The experimental results demonstrate an enhanced accuracy of sentiment recognition using this method. Wu et al. [31] proposed to construct a slang sentiment dictionary using online resources, which can effectively identify the sentiment analysis of online resources.

To some extent, the accuracy of aspect-level sentiment recognition is effectively improved by sentiment dictionary-based methods. However, as the network advances, a growing quantity of emerging words appear online, especially in cases where simple sentiment dictionaries ignore the artistic conception of the context, leading to a rapid decline in recognition accuracy.

#### 2.2.2 Deep learning-based approach

According to recent research, deep learning techniques surpass conventional machine learning methods in terms of performance in the ABSA (Aspect-Based Sentiment Analysis) classification duty. Such as, Xu et al. [32] recommended a novel algorithm that leverages gating CNN to handle the correspondence between sentence and aspect words. Specifically, they designed one gating *Tanh* CNN and another gating *RuLE* CNN to handle this task separately. Similarly, A hybrid model comprising RNNs and CRFs was proposed by Wang et al. [33], to learn features that can effectively discriminate between classes and bidirectional propagation information of aspect words and their corresponding opinion words. This framework outperforms other baseline framework on the SemEval2014 dataset.

LSTM is a classical model for handling sentiment analysis of aspect-level texts, as it can handle both long short-term dependencies. Tang et al. [34] recommended a TD-LSTM network was developed using target dependence as a basis, which divided the text into two parts with the center of aspect words, and achieved good results by inputting one part into the LSTM network in a positive order and the other part in reverse order, building on the TD-LSTM network, TC-LSTM network was developed and demonstrated improved performance. Jelodar et al. [35] applied the LSTM network to analyze aspect words in the context of COVID-19, contributing to decision-making in COVID-19 prevention and treatment.

The introduction of attention mechanisms further improved the precision of the sentiment recognition at an aspect-level. A LSTM model incorporating attention and aspect embedding was recommended by Wang et al. [22], which forced aspect to conduct an attention vector and focused on crucial segments of the text. This approach achieved good outcomes regarding the SemEval2014 dataset, demonstrating the efficacy of utilizing attention mechanisms in ABSA. Liu et al. [36] extended the attention model by distinguishing between the fine-grained left and right context and focusing on the contribution of each word to aspect, resulting in significantly improved performance on the T-Dataset and Z-Dataset datasets. Yang et al. [37] recommend an alternating coattention network, which can alternately model aspect-level and context-level attention, enabling effective feature learning. The traditional baseline model with attention mechanism was outperformed by this network on the SemEval2014 dataset, providing a new research direction for aspect-level sentiment recognition. To address the ASTE problem, Peng et al. [38] proposed a two-step assembly line that predicts aspect and corresponding opinions in the text in the first step and matches the results with sentiment trends in the second step, using triples (aspect word, corresponding opinions, corresponding sentiment trend). This approach promotes the sentimental classification of aspect-level. Graph convolutional neural networks (GCNs) [39] and BERT [40] models have exhibited remarkable performance in text and sentiment classification. For instance, Wang et al. [41] proposed the KGBGCN model, which effectively addresses the challenge of capturing key information in lengthy documents and overcoming the deficiency in classification accuracy due to the lack of domain-specific knowledge. Additionally, Xiao et al. [42] introduced the BERT4GCN model, which integrates the grammatical sequential features from BERT’s pre-trained language model (PLM) with syntactic knowledge derived from dependency graphs. This model has yielded exceptional results in aspect-based sentiment classification (ABSC) tasks. Liang et al. [43] proposed a new solution that constructs graphs using dependency trees and common-sense knowledge of sentiment. This solution utilizes graph convolutional networks to capture sentiment dependencies corresponding to specific aspects.

## 3 Framework construction

**Fig 1:**
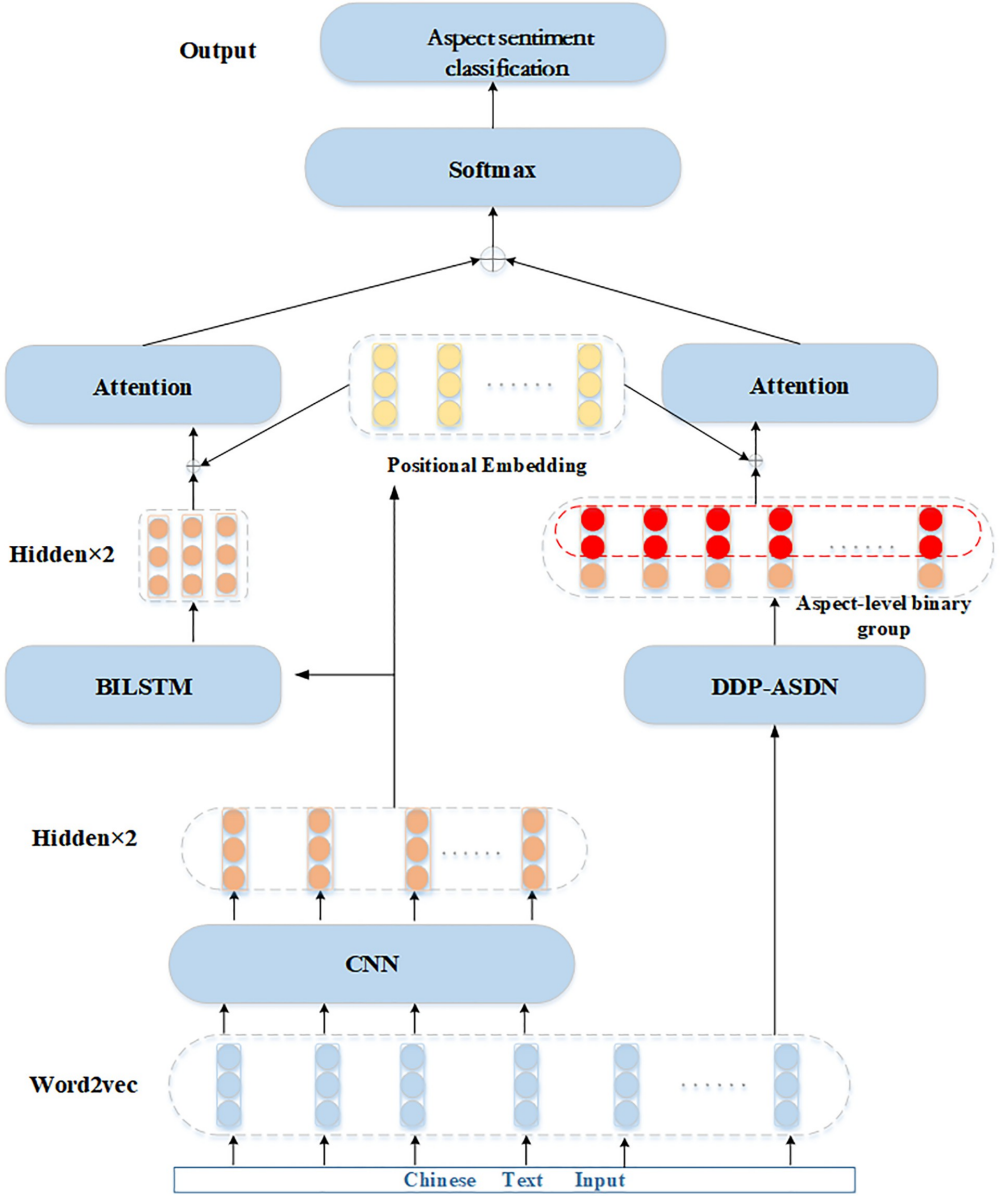
The framework Of ASN-CSD.

ASN-CSD: The framework is constructed for the aspect-level sentiment classification in this research. In addition, the framework diagram is presented in Fig 1.

An approach for aspect-level sentiment recognition, which consists of six stages, is proposed in this research, which are outlined as follows:

Step 1: The input text is encoded using a pre-trained Word2vec model. The text is divided into two categories: short text that is directly encoded into word vectors and long text that first needs to identify sentences and then encode sentence-level vectors.
Step 2: The framework uses a CNN to calculate the input text vector matrix and extract main features. Two hidden layers are used to facilitate backpropagation for parameter update. Additionally, a newly designed DDP-ASDN algorithm model is employed to extract aspect-level sentiment binary groups based on sentiment dictionary internal aspect-level syntax dependence.
Step 3: The main features extracted by the CNN model are further processed using a BILSTM model to analyze sentences with bidirectional dependencies. Two hidden layers are used to facilitate backpropagation for parameter update.
Step 4: Position coding is added to the results of the previous step to obtain a more precise position relationship between the whole and local sentences.
Step 5: Attention mechanisms are employed to give attention to the relationship surrounded by the whole context level and aspect-level, and merge the two levels.
Step 6: The outputs of the preceding stage are normalized, and sentiment recognition at the aspect-level is derived.

**Fig 2:**
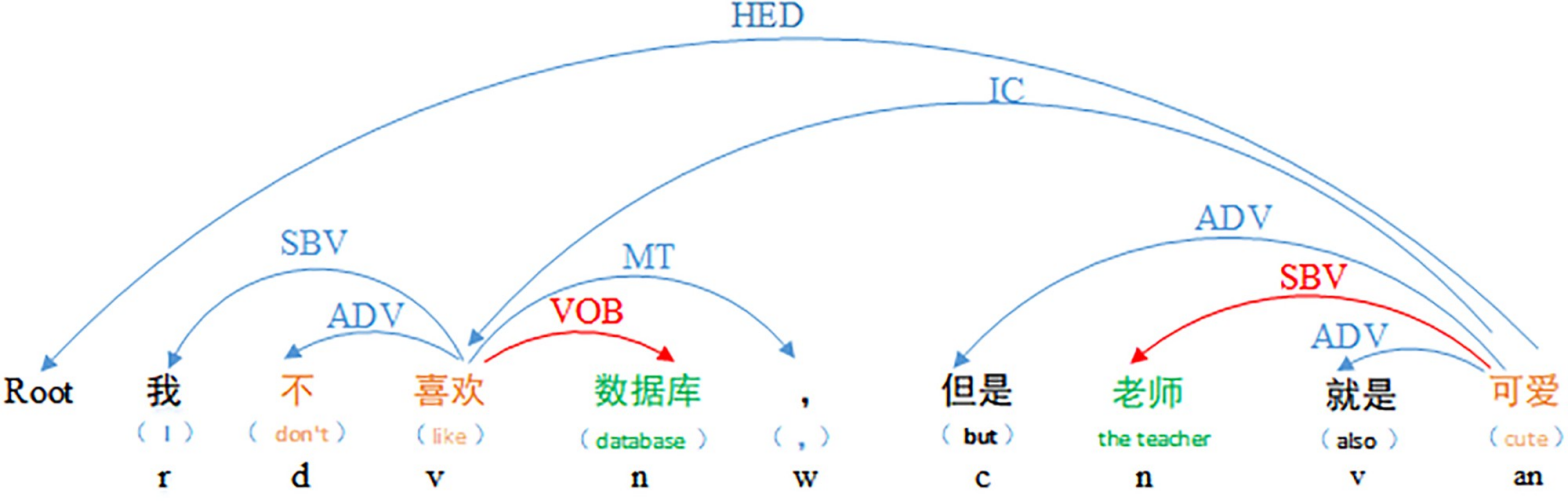
Aspect-level sentiment recognition.

A case of aspect-level sentiment recognition is presented in Fig 2. This study presents a case example using a Chinese short text “I don’t like database, but the teacher is cute.” to demonstrate the syntactic dependency relationship between aspect-level sentiment words and their corresponding sentiment options, construct sentiment binary tuples, and ultimately obtain sentiment ternary tuples with sentimental tendencies through analysis.

### 3.1 Short text and long text sentence recognition

To facilitate Natural Language Processing of short text *T* and summary equal-length text *LT* in the context of online education, a method is proposeed to convert them into word-level features. Specifically, for short text *T*, it is identified as a statement and converted into *T*_*word*_, which consists of *n* words. For *LT*, which is typically longer and contains multiple sentences, compartments are created based on punctuation marks such as commas, periods, and each compartment is then converted into a sentence-level feature *LT*_*sentence*_ containing *n* words. Thus, *LT*_*word*_ is composed of *m* such sentence-level features. This method provides a useful tool for analyzing and processing natural language text in online education.

$$\begin{array}{c} T_{word}=\{w_{1},w_{2},w_{3},\cdots ,w_{n}\} \end{array}$$ (1)

$$\begin{array}{c} LT_{word}=\{\begin{array}{c} {w_{1}}^{\left(1\right)},{w_{2}}^{\left(1\right)},{w_{2}}^{\left(1\right)},\ldots ,{w_{n}}^{\left(1\right)}\\ {w_{1}}^{\left(2\right)},{w_{2}}^{\left(2\right)},{w_{2}}^{\left(2\right)},\ldots ,{w_{n}}^{\left(2\right)}\\ {w_{1}}^{\left(3\right)},{w_{2}}^{\left(3\right)},{w_{2}}^{\left(3\right)},\ldots ,{w_{n}}^{\left(3\right)}\\ \ldots \\ {w_{1}}^{\left(m\right)},{w_{2}}^{\left(m\right)},{w_{2}}^{\left(m\right)},\ldots ,{w_{n}}^{\left(m\right)} \end{array}\} \end{array}$$ (2)

### 3.2 Acquisition of embedding vector Word2vec

Word2vec is a widely used pre-trained model for mapping words to vectors and measuring the distance and relationship between them. In this study, the skip-gram method of Word2vec is utilized to generate embedding vectors for the word-level features *T*_*word*_ and *LT*_*word*_. Specifically, $V_{word}\varepsilon R^{n\times z}$ and $LV_{word}\varepsilon R^{m\times n\times z}$ are obtained by mapping each word in *T*_*word*_ and *LT*_*word*_ to a z-dimensional vector using the trained Word2vec model.

By leveraging the semantic information captured in the Word2vec vectors. An effective approach for analyzing and processing natural language text in the context of online education is provided by this method. A variety of applications can use this embedding vectors, such as measuring similarity between words and identifying relevant concepts in the text.

$$\begin{array}{c} V^{word}=\{v_{w1},v_{w2},v_{w3},\cdots ,v_{wn}\} \end{array}$$ (3)

$$LV_{word}=\left\{\begin{array}{c} {v_{w1}}^{\left(1\right)},{v_{w2}}^{\left(1\right)},{v_{w3}}^{\left(1\right)},\cdots ,{v_{wn}}^{\left(1\right)}\\ {v_{w1}}^{\left(2\right)},{v_{w2}}^{\left(2\right)},{v_{w3}}^{\left(2\right)},\cdots ,{v_{wn}}^{\left(2\right)}\\ {v_{w1}}^{\left(3\right)},{v_{w2}}^{\left(3\right)},{v_{w3}}^{\left(3\right)},\cdots ,{v_{wn}}^{\left(3\right)}\\ \cdots \\ {v_{w1}}^{\left(m\right)},{v_{w2}}^{\left(m\right)},{v_{w3}}^{\left(m\right)},\cdots ,{v_{wn}}^{\left(m\right)} \end{array}\right\}$$ (4)

### 3.3 Aspect-level DDP-ASDN model construction

**Fig 3:**
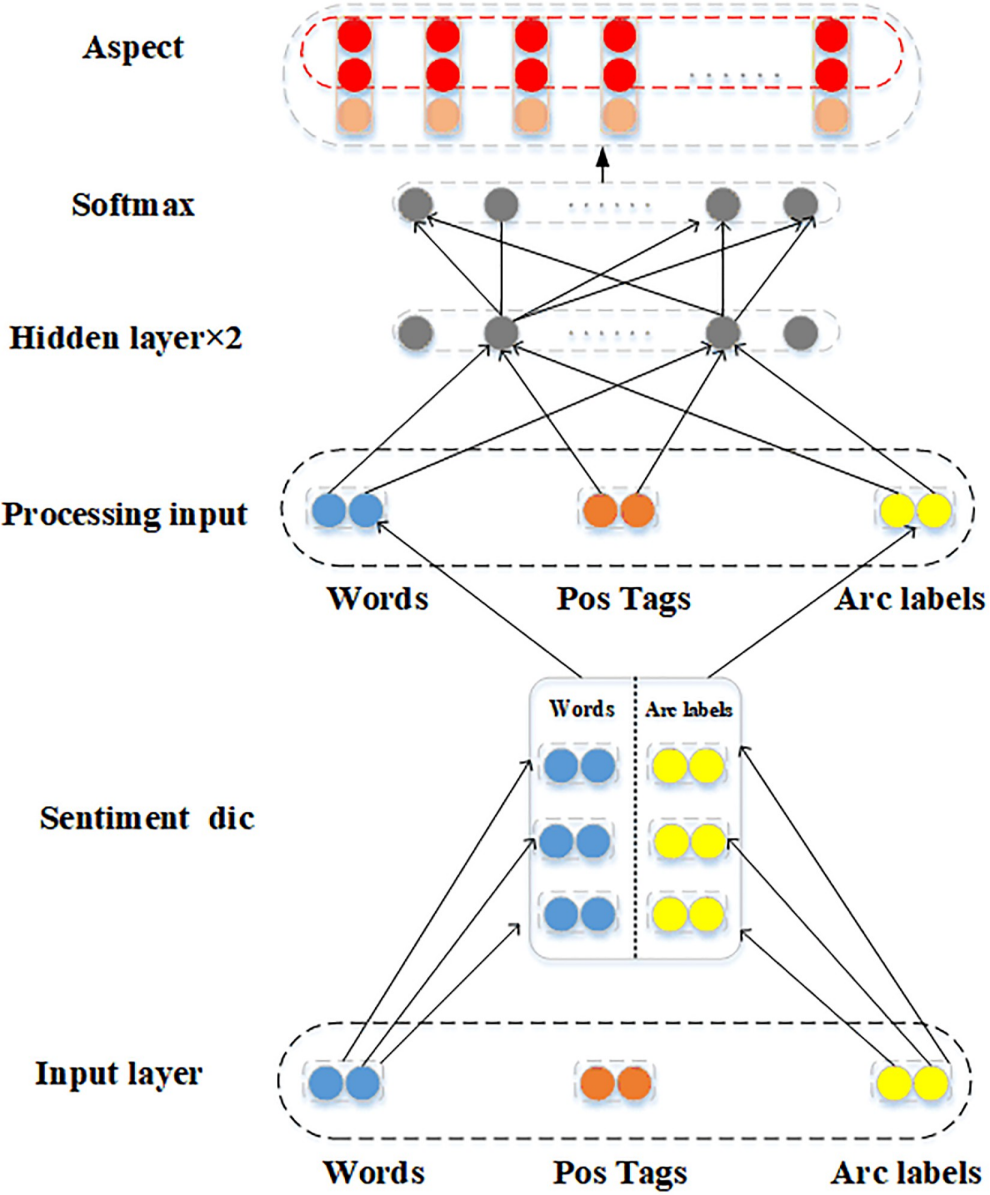
Sentence dependence analysis.

Sentence dependency refers to the dependency between words within a sentence. Label, part of speech, and location information of words can be calculated through dependency analysis. By syntactic analysis of the input text, pairs of associated words and long-distance dependent word equivalence can be obtained, making it one of the commonly used methods in sentiment analysis tasks. As an example, consider the following sentence, “I don’t like database, but the teacher is also cute.” a dependency analysis is shown in Fig 3. The relationship types between words are mostly related to nouns, and the evaluation object, “database,” and its corresponding sentimental polarity, “don’t like,” can be extracted based on the nature of words.

**Fig 4:**
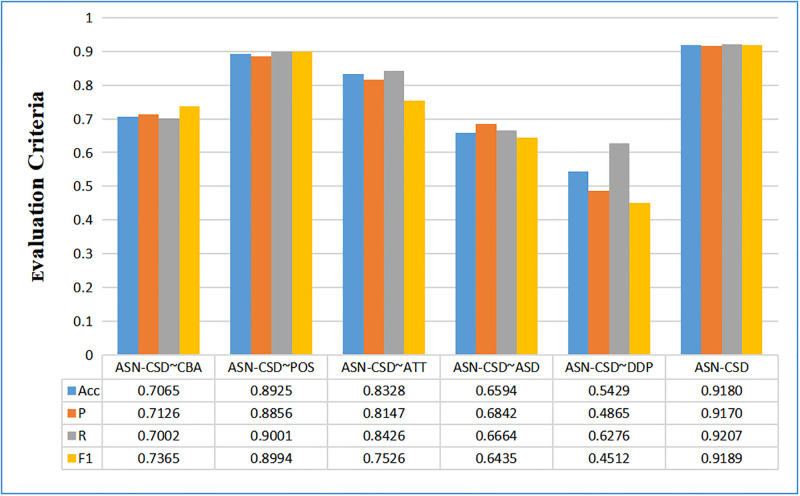
DDP-ASDN model architecture diagram.

The sentiment dictionary collects commonly used sentimental words and their corresponding label. The sentence dependency analysis model, which is added with the sentiment dictionary, can directly detect the correlation of sentimental words in text. In addition, the processing process of other unrelated words can be ignored. The framework consists of six layers. In the first layer, the incoming parameter *V*_*word*_ and *LV*_*word*_ are required to calculate the position *p* of the word and its corresponding label *L*. The second layer is the sentiment dictionary layer, where *V*_*word*_ and *LV*_*word*_ are compared with the pre-constructed sentimental dictionary. In the third layer, if *V*_*word*_ and *LV*_*word*_ belong to the sentimental dictionary, a new sentiment evaluation object $\tilde{T_{word}}$ and its corresponding sentiment evaluation label $\tilde{T_{word}^{l}}$ are built. In the fourth layer, two fully connected operations are performed to obtain *h*. In the fifth layer, a normalization operation is done to obtain $\tilde{T_{word}^{h}}$ using the softmax function. The sixth layer is the aspect layer, where a new aspect-level binary group $\tilde{T_{word}^{l}}$ is obtained by calculation. The architecture of the DDP-ASDN model is exhibited in Fig 4.

The processing process of the DDP-ASDN model is outlined in Algorithm 1:

**Algorithm 1**: DDP-ASDN

**Input**: input parameters *V*_*word*_, *LV*_*word*_

**Output**: aspect-level binary group

**1** some description;

**2 for** *Until the last sentence* **do**

**3**  Calculate the position *p* of the word and its corresponding label *L*;

**4**  *P* = {*p*^*w*1^, *p*^*w*1^, *p*^*w*1^, ⋯, *p*^*wn*^};

**5**  *L* = {*l*^*w*1^, *l*^*w*1^, *l*^*w*1^, ⋯, *l*^*wn*^};

**6**  Find a sentiment dictionary;

**7**  *Dic* = {*W*^*k*1^, *W*^*k*2^, *W*^*k*3^, ⋯, *W*^*kz*^};

**8**  *Dic*^*l*^ = {*W*^*l*1^, *W*^*l*2^, *W*^*l*3^, ⋯, *W*^*lz*^};

**9**  **if** *V*_*word*_, *LV*_*word*_ *in Dic* **then**

**10**   Construct a new sentiment evaluation object;

**11** $\tilde{T_{word}}=\{\tilde{w1},\tilde{w2},\tilde{w3},\cdots ,\tilde{wk}\}$ ;

**12**  Construct the corresponding sentiment evaluation labels;

**13** $\tilde{T_{word}^{l}}=\{\tilde{w1^{l}},\tilde{w2^{l}},\tilde{w3^{l}},\cdots ,\tilde{wk^{l}}\}$ ;

**14**  Enter the hidden layer for calculation;

**15** $h=(W_{1}^{\tilde{w1}}\tilde{w1}+W_{2}^{\tilde{w2}}\tilde{w2}+W_{3}\tilde{w3}\tilde{w3}+\cdots +W_{k}^{\tilde{wk}}\tilde{wK}+b_{1}{)}^{2}$ ;

**16**  Enter the softmax for the calculation;

**17** $\tilde{T_{word}^{h}}=softmax\left(W_{2}h\right)$ ;

**18**  Combined into a new aspect-level binary group;

**19** $\tilde{T_{word}^{l}}=\{\left(\tilde{w_{1}^{h}},\tilde{l1}\right),\left(\tilde{w_{2}^{h}},\tilde{l2}\right),\left(\tilde{w_{3}^{h}},\tilde{l3}\right),\cdots ,\left(\tilde{w_{k}^{h}},\tilde{lk}\right)\}$ ;

**20**  **end**

**21** **end**

### 3.4 CNN-BILSTM-ATTENTION model construction

The main features of the input vector are obtained through CNN, and are taken as the input for subsequent calculations to reduce the amount of computing resources required for the entire algorithm. Next, the input is passed through the BILSTM layer to collect the long short-term associations between words in the sentence. The following five steps can be used to describe the specific processing procedure.

Step 1: The eigenvalues $C_{i}^{V^{word}}$ and $C_{i}^{LV^{word}}$ were computed by the CNN model’s convolution layers for the incoming *V*_*word*_ and *LV*_*word*_, respectively. The computation process is defined as follows:

$$\begin{array}{c} C_{i}^{V^{word}}=Relu\left(W_{word}^{CNN}V^{word}+b_{word}^{CNN}\right)\;\;i\varepsilon \{1,2,\cdots ,s\} \end{array}$$ (5)

$$\begin{array}{c} C_{i}^{LV^{word}}=Relu\left(W_{word}^{CNN}LV^{word}+b_{word}^{CNN}\right)\;\;i\varepsilon \{1,2,\cdots ,s\} \end{array}$$ (6)

*Relu* is the activation function, $W_{word}^{CNN}$ refers to the weight, while $b_{word}^{CNN}$ corresponds to the bias term, and *i* is the index for the quantity of convolution kernels. Then, the convolution layer results $C_{i}^{V^{word}}$ and $C_{i}^{LV^{word}}$ are passed into the maximum pooling layer *Max*() of the CNN model to calculate ${\hat{C}}^{V^{word}}$ and ${\hat{C}}^{LV^{word}}$. Finally, the results are passed through two fully connected layers, which are defined as follows:

$$\begin{array}{c} {\hat{C}}^{V^{word}}=Max\left(C_{i}^{V^{word}}\right)\;\;i\varepsilon \{1,2,\cdots ,s\} \end{array}$$ (7)

$$\begin{array}{c} {\hat{C}}^{LV^{word}}=Max\left(C_{i}^{LV^{word}}\right)\;\;i\varepsilon \{1,2,\cdots ,s\} \end{array}$$ (8)

Step 2: ${\hat{C}}^{V^{word}}$ and ${\hat{C}}^{LV^{word}}$ are fed into the BILSTM layer separately to obtain $\vec{h_{t}^{V}}$ and $\vec{h_{t}^{LV}}$ through forward propagation, followed by reverse propagation to obtain $\vec{h_{t}^{LV}}$ and $\overset{\leftarrow }{h_{t}^{LV}}$. Finally, the forward and reverse propagation results of the short text are added to obtain $h_{t}^{V}$, and the forward and reverse propagation results of the long text are added to obtain $h_{t}^{LV}$. which $\vec{V}$ and $\overset{\leftarrow }{V}$ represent hidden layer trainable parameters,$\vec{w}$ and $\overset{\leftarrow }{w}$ represent input layer trainable parameters, $\vec{b}$ and $\overset{\leftarrow }{b}$ represent bias, ⊕ represents the concatenation of vectors.

$$\begin{array}{c} \vec{h_{t}^{V}}=f\left(\vec{V}*{\vec{h}}_{t-1}+\vec{W}*{\hat{C}}_{t}^{V^{word}}+\vec{b}\right) \end{array}$$ (9)

$$\begin{array}{c} \vec{h_{t}^{LV}}=f\left(\vec{V}*{\vec{h}}_{t-1}+\vec{W}*{\hat{C}}_{t}^{LV^{word}}+\vec{b}\right) \end{array}$$ (10)

$$\begin{array}{c} \overset{\leftarrow }{h_{t}^{V}}=f\left(\overset{\leftarrow }{V}*{\overset{\leftarrow }{h}}_{t+1}+\overset{\leftarrow }{W}*{\hat{C}}_{t}^{V^{word}}+\overset{\leftarrow }{b}\right) \end{array}$$ (11)

$$\begin{array}{c} \overset{\leftarrow }{h_{t}^{LV}}=f\left(\overset{\leftarrow }{V}*{\overset{\leftarrow }{h}}_{t+1}+\overset{\leftarrow }{W}*{\hat{C}}_{t}^{LV^{word}}+\overset{\leftarrow }{b}\right) \end{array}$$ (12)

$$\begin{array}{c} h_{t}^{V}=\vec{h_{t}^{V}}⊕\overset{\leftarrow }{h_{t}^{V}} \end{array}$$ (13)

$$\begin{array}{c} h_{t}^{LV}=\vec{h_{t}^{LV}}⊕\overset{\leftarrow }{h_{t}^{LV}} \end{array}$$ (14)

Position coding(*Pe*) is added after the BILSTM layer to effectively incorporate location information. First, the output of the CNN layer, ${\hat{C}}^{V^{word}}$ and ${\hat{C}}^{LV^{word}}$, and $\tilde{T_{word}^{h}}$ are fed into the position coding layer to obtain corresponding word position information. The location information is encoded using the transformer and reference in the position coding method. The corresponding position-coding is calculated as follows:

$$\begin{array}{c} \begin{array}{c} Pe\left(pos_{{\hat{C}}^{V^{word}}},2i\right)=sin(\frac{pos_{{\hat{C}}^{V^{word}}}}{10000\frac{2i}{z}})\\ TPe\left(pos_{{\hat{C}}^{LV^{word}}},2i\right)=sin(\frac{pos_{{\hat{C}}^{LV^{word}}}}{10000\frac{2i}{z}})\\ Pe\left(pos_{{\hat{C}}^{V^{word}}},2i+1\right)=cos(\frac{pos_{{\hat{C}}^{V^{word}}}}{10000\frac{2i}{z}})\\ Pe\left(pos_{{\hat{C}}^{LV^{word}}},2i+1\right)=cos(\frac{pos_{{\hat{C}}^{LV^{word}}}}{10000\frac{2i}{z}})\\ Pe\left(pos_{T_{word}^{h}},2i\right)=sin(\frac{pos_{T_{word}^{h}}}{10000\frac{2i}{z}})\\ Pe\left(pos_{T_{word}^{h}},2i+1\right)=cos(\frac{pos_{T_{word}^{h}}}{10000\frac{2i}{z}}) \end{array} \end{array}$$ (15)

The placement of each word in relation to others in the short text is denoted as $pos_{{\hat{C}}^{V^{word}}}$, while the set of relative positions of each word in the long text is represented as $pos_{{\hat{C}}^{LV^{word}}}$. Similarly, the set of relative positions of each word in the aspect-level after the DDP-ASDN model is represented as $pos_{T_{word}^{h}}$, and *z* denotes the embedding dimension. The phase position coding of each word is combined into a new *z*-dimension matrix using the sine cosine function. The combined output matrix of short text, long text, and aspect-level is given as follows:

$$\begin{array}{c} \begin{array}{c} Pe\left(pos_{{\hat{C}}^{V^{word}}}\right)=\{Pe\left(pos_{{\hat{C}}^{V^{word}}},2i\right),Pe\left(pos_{{\hat{C}}^{V^{word}}},2i+1\right),\cdots ,Pe\left(pos_{{\hat{C}}^{V^{word}}},z\right)\}\\ Pe\left(pos_{{\hat{C}}^{LV^{word}}}\right)=\{Pe\left(pos_{{\hat{C}}^{LV^{word}}},2i\right),Pe\left(pos_{{\hat{C}}^{LV^{word}}},2i+1\right),\cdots ,Pe\left(pos_{{\hat{C}}^{LV^{word}}},z\right)\}\\ Pe\left(pos_{T_{word}^{h}}\right)=\{Pe\left(pos_{T_{word}^{h}},2i\right),Pe\left(pos_{T_{word}^{h}},2i+1\right),\cdots ,Pe\left(pos_{T_{word}^{h}},z\right)\} \end{array} \end{array}$$ (16)

Step 4: The results of position coding, $Pe(pos_{{\hat{C}}^{V^{word}}})$, $Pe(pos_{{\hat{C}}^{LV^{word}}})$, and $Pe(pos_{T_{word}^{h}})$, are added to the results of the BILSTM layer, $h_{t}^{V}$ and $h_{t}^{LV}$, to obtain new encoded representations,short text $h_{pe}^{V}$ and long text $h_{pe}^{LV}$.

$$\begin{array}{c} \begin{array}{c} h_{pe}^{V}=h_{t}^{V}+Pe\left(pos_{{\hat{C}}^{V^{word}}}\right)\\ h_{pe}^{LV}=h_{t}^{LV}+Pe\left(pos_{{\hat{C}}^{LV^{word}}}\right) \end{array} \end{array}$$ (17)

Step 5: The encoded representations, $h_{pe}^{V}$, $h_{pe}^{LV}$ and sentiment evaluation label $\tilde{T_{word}^{l}}$ are used as input to the normalization layer, to obtain new short text encoding *h*^*V*^, long text encoding *h*^*LV*^ and sentiment evaluation label enconding *T*^*DDP*^. which performs the following steps:

$$\begin{array}{c} \begin{array}{c} h^{V}=softmax\left(h_{pe}^{V}\right)\\ h^{LV}=softmax\left(h_{pe}^{V}\right)\\ T^{DDP}=softmax\left(\tilde{T_{word}^{l}}\right) \end{array} \end{array}$$ (18)

The impact factors are then computed based on the normalized results, which are defined as follows. The symbol *a* represents the ATTENTION mechanism, which calculates the attention between different words.

$$\begin{array}{c} \begin{array}{c} e_{ij}^{V}=a\left(h_{i-1}^{V},h_{j}\right)\\ e_{ij}^{LV}=a\left(h_{i-1}^{LV},h_{j}\right)\\ e_{ij}^{DDP}=a\left(T_{i-1}^{DDP},h_{j}\right) \end{array} \end{array}$$ (19)

The weight distribution of the attentions are obtained by calculating the influence factor, defined as follows:

$$\begin{array}{c} \begin{array}{c} a_{ij}^{V}=\frac{exp(e_{ij})}{\sum_{k=1}^{n}exp\left(e_{ik}\right)}\\ a_{ij}^{LV}=\frac{exp(e_{ij})}{\sum_{k=1}^{n}exp\left(e_{ik}\right)}\\ a_{ij}^{DDP}=\frac{exp(e_{ij})}{\sum_{k=1}^{k}exp\left(e_{ik}\right)} \end{array} \end{array}$$ (20)

Then the weight factors are summed over, which can be defined as follows:

$$\begin{array}{c} \begin{array}{c} c_{i}^{V}=\sum_{j=1}^{n}a_{ij}^{V}h_{j}\\ c_{i}^{LV}=\sum_{j=1}^{n}a_{ij}^{LV}h_{j}\\ c_{i}^{DDP}=\sum_{j=1}^{k}a_{ij}^{DDP}h_{j} \end{array} \end{array}$$ (21)

Then the attention results of the two layers are summed to obtain the attention that contains the context and aspect-level relationships. This is defined as follows:

$$\begin{array}{c} Att_{contex}^{DDP}=c_{i}^{V}+c_{i}^{LV}+c_{i}^{DDP} \end{array}$$ (22)

### 3.5 Classification results

The attention vector $Att_{context}^{DDP}$ undergoes normalization after being processed by a fully connected layer, and the classification label is obtained by taking the maximum value of the normalized result. Finally, the aspect-level *Triples* are combined for each aspect sentiment recognition ensemble. which *W*^*Att*^ represents the trainable parameters of the dual attention layer.

$$\begin{array}{c} label=softmax(Att_{contex}^{DDP}W^{Att}) \end{array}$$ (23)

$$\begin{array}{c} Triples=\{\left(\tilde{w_{1}^{h}},\tilde{l1},pos\right),\left(\tilde{w_{2}^{h}},\tilde{l2},neg\right),\left(\tilde{w_{3}^{h}},\tilde{l3},neu\right),\cdots ,\left(\tilde{w_{k}^{h}},\tilde{lk},pos\right)\} \end{array}$$ (24)

## 4 Experiment

### 4.1 Datasets

Two publicly available datasets and one collected by our team were selected in this paper. The detailed descriptions of the datasets are provided below.

1: The Fine-grained Automotive Comment Standard Dataset (dataset 1), comprises 56,920 comments collected from various automotive forums. User comments were annotated with labels such as manufacturer, brand, model, attribute, descriptive value, tendency, and more. This dataset can be download from: https://www.datatang.com/dataset/info/text/135
2: The Tan Songbo-Hotel Review Corpus (dataset 2), consists of 10,000 articles automatically collected and collated from Ctrip. The corpus comprises 7,000 positive sentiment data and 3,000 negative sentiment data. This dataset can be download from: https://pan.baidu.com/s/1TrumHVMk-Kc4PJz8INMYbg
3: The U+ Wisdom Teaching Platform Dataset (dataset 3), was extracted from the U+ New Engineering Wisdom Cloud Platform. The dataset includes course evaluation data and experimental summaries of 50 universities, comprising a total of 9,000 long texts and 12,000 short texts. The dataset contains two sentiment categories: positive and negative, which can be further decomposed into fine-grained sentiment, totaling 30,000. This dataset can be download from: https://www.eec-cn.com

All experiments were conducted on four Nvidia V100 GPUs, and the datasets were split into a ratio of 6:1:3 for training, validation, and testing sets.

### 4.2 Baseline methods

In order to assess the efficacy of the recommended techniques, a comparison is made with the ten most advanced methods as described below:

ATAE-LSTM [44]: The sentences are processed based on the LSTM model, weighted and aggregated by combining the association of context and aspect terms.

BiGCN [45]: A graph convolutional network with two levels of interaction is devised based on dependency tree and word co-occurrence relationships to fully learn the node representation.

TNet [46]: The sentence feature representation is encoded using BILSTM and undergoes continuous aspect-level context encoding and attention mechanism, and the final feature representation is extracted using CNN.

DualGCN [47]: The SemGCN two-channel structure integrates grammar knowledge and semantic information with SynGCN and attention mechanism based on the dependent analytical probability matrix. Orthogonal regularization and differential regularization in SemGCN help the framework to grasp meaning associations different from the grammatical structure.

MemNet [11]: Context sentences are considered as external memory and attention mechanism with multiple heads are applied to the word vector characterization of the context. The final aspect characterization is derived from the output of the last hop.

DM-GCN [48]: Syntactic graph and semantic graph are constructed based on dependent tree and multi-head self-attention mechanism respectively. Information is extracted through syntactic graph convolution (Syntax GCN) and semantic graph convolution (Semantic GCN) respectively. The Common GCN module is utilized with a parameter sharing strategy to acquire shared information from the two spaces. The information extracted from the three channels is fused and used for the classification task.

ASGCN [49]: Feature representations of sentences are acquired using BILSTM. Aspect-level context representations are learned through dependency tree-based GCN, and aggregated context representations are used for classification using the attention mechanism.

GTS [50]: A grid marking scheme is designed to solve the triplet separation problem in an end-to-end system. The adopted inference strategy makes full use of the indicating role between different opinion elements.

IMN+IOG [51]: The interactive network IMN is used to extract explicit target aspects in the sentence. Then, the IOG framework is used to derive the associated opinion words to generate the final triples.

IAN [52]: Two LSTM models are used to encode text environment and aspects. Additionally, the Interdependent attention mechanism is employed to model the relationships between them.

BERT [53]: The model is fine-tuned by simultaneously inputting modified new tokens as both [cls] and [seq].

RoBERTa [54]: RoBERTa is built on the basis of the language masking strategy in BERT, modifying key hyperparameters in BERT, including deleting the next sentence training objective in BERT, and using a larger batch size and learning rate for training. It is relatively friendly to Chinese text classification.

dotGCN [55]: This model introduced a discrete latent tree, a structure designed to serve as a substitute for the traditional dependency tree. This new structure is language-independent and specifically tailored to a particular aspect.

### 4.3 Evaluation criteria

The evaluation metrics employed in this article consist of Accuracy(Acc), Precision(P), Recall(R), and Macro-F1(F1). The definitions of the indicators are provided below [56]:

$$\begin{array}{c} \begin{array}{c} Acc=\frac{TP+TN}{TP+FP+TN+FN}\\ P=\frac{TP}{TP+FP}\\ R=\frac{TP}{TP+FN}\\ F1=\frac{2*P*R}{P+R} \end{array} \end{array}$$ (25)

*TP* stands for the true positive class, which is the case when an instance is a positive class and is predicted as such. The true negative class is denoted by *TN*, this occurs when an instance is classified as a negative class and is indeed negative. *FP* represents the false positive class, this happens when an instance is actually positive, but is classified as negative by the prediction model. The false negative class is denoted by *FN*, this is the scenario where an instance is falsely classified as negative despite being positive.

### 4.4 Experimental environment

In this paper, deep learning model is constructed based on Word2vec, CNN, BILSTM, POS (Position Coding), DDP-ASDN, ATTENTION, and Aspect-Level sentiment dictionaries. The parameter configurations are presented in Table 1:

**Table 1:** ASN-CSD model hyperparameter details.

Parameter	Value	Parameter	Value	Parameter	Value
Doc_Size	150	learning_rate	1e-5	features	2
WordVec_Size	768	Epsilon	1e-8	n_layers	2
WeightDecay	1e-5	dropout_rate	0.1	bidirect	True
epoches	200	seed	1	cuda	True
batch_size	48	hidden_dim(z)	768	num_heads	4
dim_k	768	Filter_Num	200	KMax	1
dim_v	768	Filter_size	[1, 2, 4]	Channel_Size	1

### 4.5 Experimental results

In this paper, the performance of the recommended ASN-CSD framework and ten baseline models are compared on three datasets to evaluate ABSA. The results are presented in Table 2 for the Fine-grained automotive comment standard dataset. Table 3 for the Tan Songbo-Hotel review corpus, and Table 4 for the U+ wisdom teaching platform dataset. It is experimentally demonstrated that the ASN-CSD model performed optimally on all three datasets, yielding the highest Accuracy performance, Precision, Recall, Macro-F1, and computational time. The suboptimal models are outperformed by the proposed model by approximately 0.1000 on all evaluation indices. The superior performance of the recommended framework can be come down to its dual attention mechanism of context and aspect-level sentiment dependence, which enables better identification of aspect-level sentiment. Furthermore, the proposed model exhibited excellent computational efficiency, with most models improving by approximately 100.0 seconds. This is due to the use of a sentiment dictionary to pre-identify the dependence relationship and the relationship between internal words, thus reducing computation time.

**Table 2:** Classification results on dataset fine-grained automotive comment standard dataset (Optimal results are bold).

Model	Acc	P	R	F1	Time(s)
ATAE-LSTM	0.7392	0.8835	0.7158	0.7909	92.5
BiGCN	0.7453	0.8538	0.7241	0.7836	100.4
TNet	0.6569	0.6666	0.757	0.7089	225.6
DualGCN	0.7813	0.8738	0.6725	0.7601	428.5
MemNet	0.7897	0.8915	0.7579	0.8193	86.4
DM-GCN	0.7338	0.7639	0.7796	0.7717	99.6
ASGCN	0.7614	0.8353	0.7072	0.7659	214.5
GTS	0.8107	0.7729	0.8894	0.8271	368.4
IMN+IOG	0.7829	0.8574	0.7063	0.7746	352.8
IAN	0.7597	0.6941	0.8908	0.7803	469.9
BERT	0.8269	0.7354	0.8356	0.7965	200.3
RoBERTa	0.8479	0.8354	0.8476	0.7845	210.9
dotGCN	0.8487	0.8426	0.8027	0.8634	569.9
**ASN-CSD**	**0.9180**	**0.9170**	**0.9207**	**0.9189**	**82.6**

**Table 3:** Classification results on Tan Songbo-Hotel review corpus (Optimal results are bold).

Model	Acc	P	R	F1	Time(s)
ATAE-LSTM	0.6792	0.776	0.6886	0.7297	254.6
BiGCN	0.7153	0.7983	0.7105	0.7518	124.3
TNet	0.6569	0.6033	0.7032	0.6494	158.4
DualGCN	0.7513	0.8480	0.7258	0.7821	476.5
MemNet	0.7897	0.8704	0.7625	0.8129	95.6
DM-GCN	0.7138	0.7300	0.7717	0.7502	153.6
ASGCN	0.7614	0.8643	0.7521	0.8043	247.9
GTS	0.8107	0.7649	0.8849	0.8206	354.8
IMN+IOG	0.8829	0.8841	0.8957	0.8899	156.8
IAN	0.7597	0.7084	0.8972	0.7917	458.5
BERT	0.8326	0.7549	0.7853	0.8492	220.7
RoBERTa	0.8899	0.8467	0.8398	0.7468	250.2
dotGCN	0.8472	0.7869	0.8237	0.8471	650.4
**ASN-CSD**	**0.9081**	**0.8865**	**0.9417**	**0.9132**	**93.8**

**Table 4:** Classification results on the U + Wisdom teaching platform dataset (Optimal results are bold).

Model	Acc	P	R	F1	Time(s)
ATAE-LSTM	0.6692	0.7624	0.6886	0.7236	263.5
BiGCN	0.6153	0.6132	0.6534	0.6326	254.6
TNet	0.7569	0.7931	0.7570	0.7746	269.4
DualGCN	0.7613	0.7804	0.7823	0.7813	756.5
MemNet	0.6897	0.6798	0.7149	0.6969	254.6
DM-GCN	0.7138	0.6747	0.7217	0.6974	247.6
ASGCN	0.6614	0.6881	0.7072	0.6975	458.9
GTS	0.7107	0.7144	0.6804	0.6970	478.6
IMN+IOG	0.7829	0.6972	0.8713	0.7746	358.4
IAN	0.6597	0.6548	0.7007	0.6770	794.3
BERT	0.7698	0.7426	0.7598	0.8126	368.9
RoBERTa	0.8217	0.8011	0.8437	0.8045	300.4
dotGCN	0.7986	0.7872	0.8436	0.7756	660.8
**ASN-CSD**	**0.8380**	**0.8097**	**0.8786**	**0.8428**	**186.2**

After conducting further analysis of the outcomes of the experiments, a noticeable observation is that most of the models demonstrated better achievement on the two public datasets than on the U + wisdom teaching platform dataset. This can be attributed to the highly specialized nature of the data in the domestic education field and the limited coverage of the sentimental dictionary. Moreover, the involvement of students in the sentimental evaluation summary can lead to ambiguous sentimental expressions that fail to express their true feelings. Additionally, it is noted that the performance of most baseline models are inferior on the Chinese datasets compared to their English counterparts. However, the ASN-CSD model effectively handles Chinese data and performs well in specialized fields, indicating its strong generalization ability and potential for future promotion. Furthermore, it is found that the use of sentimental dictionaries and dependency models often leads to superior performance compared to other models. This underscores the advantages of combining aspect-level sentimental dictionaries, dependency relationships, and contextual mechanisms for the ABSA task.

### 4.6 Ablation experiment

In this section, five sets of juxtaposition models are created to further verify the effectiveness of each module in the ASN-CSD model, which are specifically described as follows:

ASN-CSD∼CBA: The CNN-BILSTM-ATTENTION module is removed, leaving only the DDP-ASDN and position coding modules.
ASN-CSD∼POS: The position coding module is removed, retaining the CNN-BILSTM-ATTENTION and DDP-ASDN modules.
ASN-CSD∼ATT: The dual attention mechanism is removed, retaining the CNN-BILSTM module, position coding, and DDP-ASDN modules.
ASN-CSD∼ASD: The aspect sentiment dictionary is removed, retaining the CNN-BILSTM-ATTENTION module, position coding, and syntactic dependency.
ASN-CSD∼DDP: The syntax-dependent DDP is removed, retaining the CNN-BILSTM-ATTENTION module, position coding, and sentiment dictionary.
ASN-CSD: The complete model presented in this paper, which retains the CNN-BILSTM-ATTENTION module, position coding, and DDP-ASDN module. These models are designed to compare and evaluate the performance of each module, allowing for a better understanding of the contribution of each module to the overall performance of the model.

**Fig 5:**
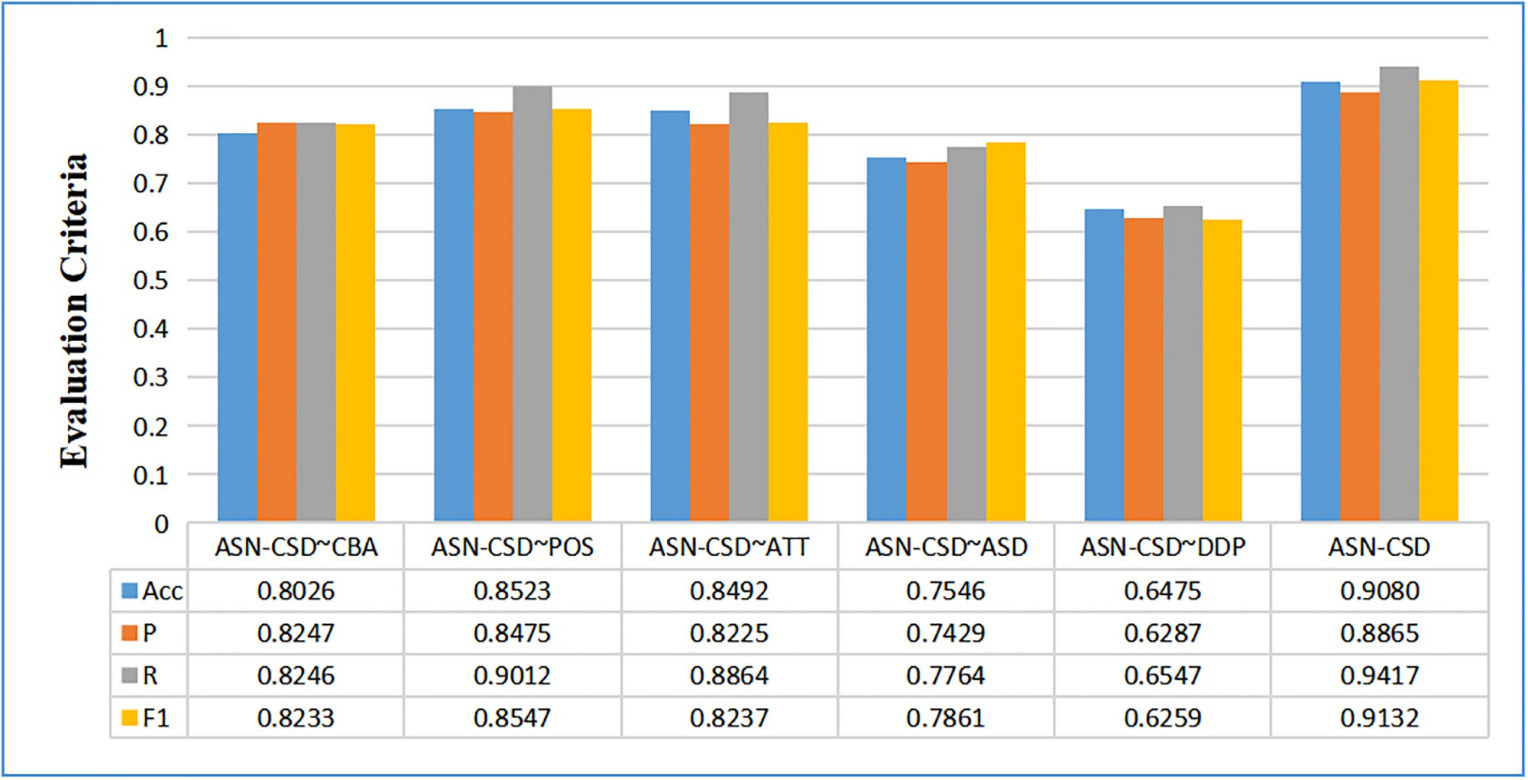
Classification results in fine-grained automotive comment standard dataset after removal of partial modules.

**Fig 6:**
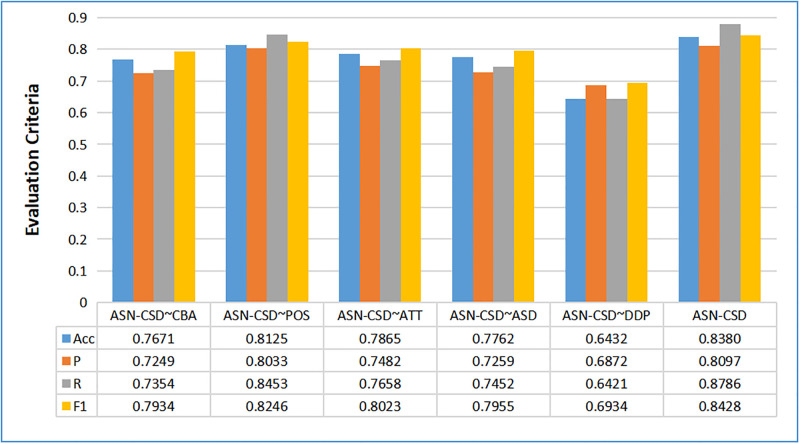
Classification results In Tan Songbo-Hotel review corpus after removal of some modules.

**Fig 7:**
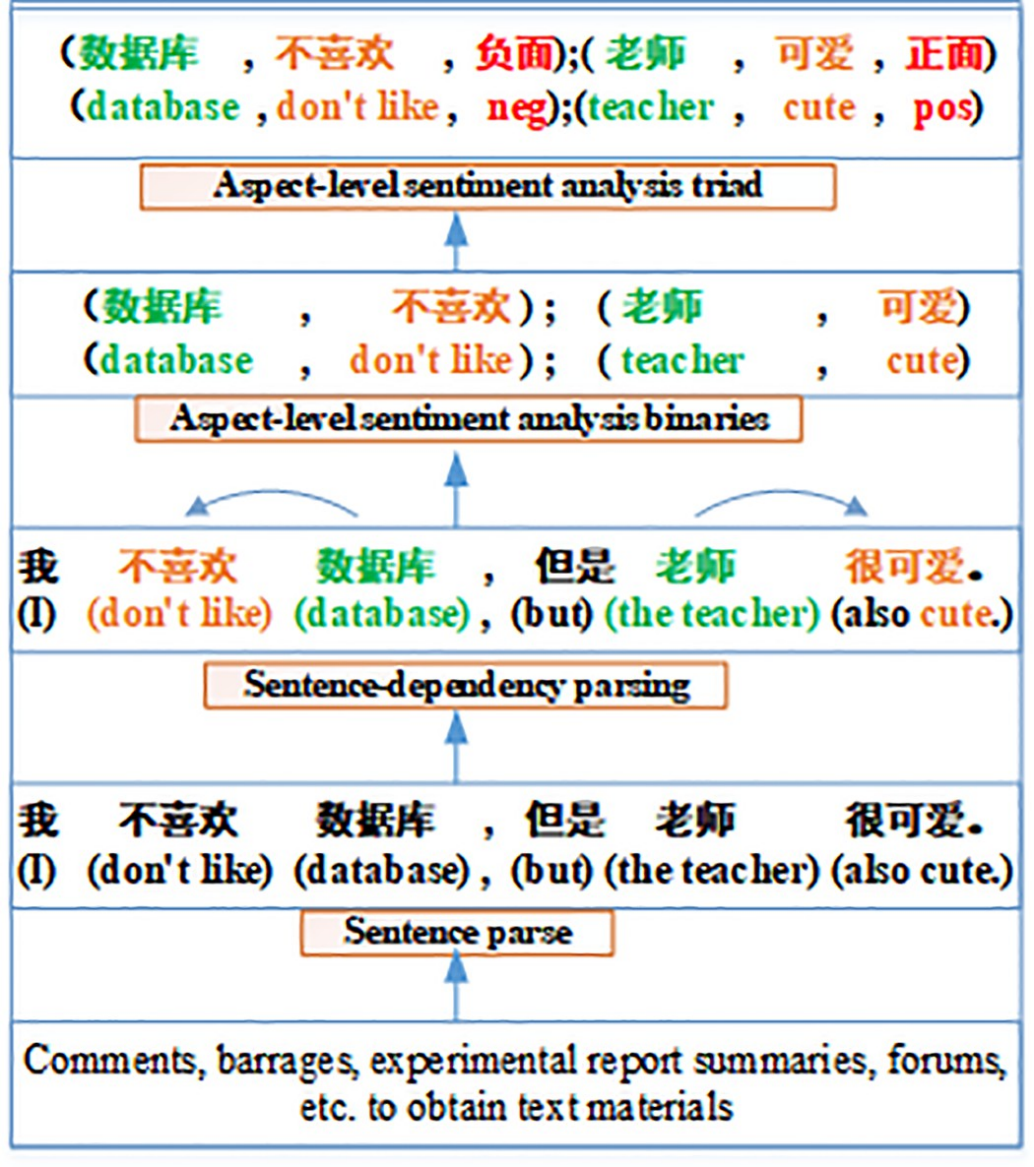
Classification results in U + Wisdom teaching platform dataset after removal of some modules.

The achievement of the five juxtaposition models on the three datasets are presented in Figs 5–7. It is observed that the removal of syntactic dependence analysis and sentiment dictionary led to a significant decline in performance, with the ASN-CSD∼DDP showing the most significant decrease, and the average accuracy decreasing to below 0.2768. This highlights the crucial role of syntactic dependence analysis and sentiment dictionary in aspect-level sentiment recognition. Sentiment dictionary can identify sentimental words, while syntactic dependence can quickly identify the sentimental tendency of these words, thus demonstrating the effectiveness of the DDP-ASDN layer presented in this paper.

The performance of the ASN-CSD∼POS and ASN-CSD∼ATT models on the three datasets reveals a decrease in performance after removing the position coding or attention mechanism. In dataset 3, the minimum drop in precision is only 0.0064, which can be attributed to the BILSTM and syntactic dependence models capturing the relationship between words within the sentence and obtaining some of the characteristics of the internal relationship.

The ASN-CSD∼CBA model shows that aspect-level sentiment classification can still be performed by removing all contextual association features and retaining only the DDP-ASDN model. However, this approach results in a decrease in performance of around 0.1000, mainly due to the lack of context-level feature vectors.

When further examining the ASN-CSD∼CBA and ASN-CSD∼ASD models on datasets 1 and 2, it is observed that the ASN-CSD∼ASD performed better than the ASN-CSD∼CBA. This result highlights the importance of the sentiment dictionary for the sentiment of recognition at an aspect-level sentiment recognition, which is more crucial than the correlation characteristics between contexts. However, on dataset 3, the ASN-CSD∼ASD model performed less effectively than the ASN-CSD∼CBA model. The reason for this may be due to the fact that the data in dataset 3 uses more professional vocabulary, leading to lower coverage of sentiment dictionaries. This result further emphasizes the significance of the proposed sentiment dictionary in aspect-level sentiment recognition.

The worst performance for the three datasets is exhibited by the ASN-CSD∼DDP model, highlighting the importance of syntactic dependence analysis in aspect-level sentiment recognition. Without dependency analysis, it is impossible to obtain the direct sentimental relationship of sentences, leading to poorer aspect-level sentiment recognition. This underscores the necessity of this paper based on syntactic dependence.

Overall, the complete ASN-CSD framework achieved the best achievement for aspect-level sentiment recognition. The ablation experiments have strongly demonstrated the necessity and effectiveness of each module in the ASN-CSD.

## 5 Conclusion

The ASN-CSD framework for aspect-level sentiment classification in Chinese short and long texts is presented in this article. The input text is encoded into word vectors using the pre-trained Word2vec model, and the aspect-level sentiment dyple is extracted by the newly designed DDP-ASDN model, which relies on a sentiment dictionary for internal aspect-level syntax. The main feature values of a sentence are extracted using CNN-BILSTM-ATTENTION, while considering the long-term and short-term dependencies and context-level feature relationships of the sentence. Additionally, the position coding is calculated to facilitate a more accurate acquisition of the aspect-level characteristics, and the double attention is applied on the context-level and aspect-level. The experiments conducted on three datasets have demonstrated the superiority of the model and the importance of each component.

However, the model’s performance can be further improved by establishing a professional Chinese sentiment dictionary and a more accurate implicit aspect-level words recognition algorithm.
